# Higher mental illness-related stigma after receiving mental health service among adolescents and youth with depressive symptoms in Chongqing, China

**DOI:** 10.3389/fpsyt.2025.1725567

**Published:** 2026-01-21

**Authors:** Yi-Hao Liu, Jian-Jun Luo, Cong Wang, Yi-Yue Yang, Lie Zhou, Hui Jin, Yun Xiao, Yang Wen, Jawad Ahmad, Michelle Yu-Xin Ran, Na Du, Jia Cai, Ji-Yuan Zhong, Mao-Sheng Ran

**Affiliations:** 1 National Center for Mental Disorders, West China Hospital, Sichuan University, Chengdu, Sichuan, China; 2 Mental Health Center, West China Hospital, Sichuan University, Chengdu, Sichuan, China; 3 Department of Social Psychiatry, West China Hospital, Sichuan University, Chengdu, Sichuan, China; 4 Chongqing Mental Health Center, Chongqing, China; 5 Chengdu Fourth People’s Hospital, Chengdu, Sichuan, China

**Keywords:** depressive symptoms, adolescents, treatment gap, mental health-related stigma, iatrogenic effect

## Abstract

**Background:**

Previous evidence suggests that some adolescent and youth students with depressive symptoms may access mental health services only once but never return for follow-up care. The current study aims to investigate whether adolescent and youth students with depressive symptoms experience increased mental illness-related stigma after receiving mental health services within the past 12 months and explore potential sex and ethnicity differences.

**Method:**

A cross-sectional online survey was conducted among adolescents and early youth attending schools in Chongqing, China (age 11–24) through convenience sampling, between November 2024 and February 2025. Participants reported their demographic information, depressive symptoms (PHQ-9), anxiety symptoms (GAD-7), mental health services received in the past 12 months, and mental illness-related stigma (PDD). Data was analysed with ANOVA and simple slope analysis.

**Results:**

In total, 33,409 students completed the survey (mean age: 16.27 ± 2.45; female = 49.6%), with 43.6% reporting depressive symptoms. Among students with depressive symptoms (mean age: 15.95 ± 2.21; female = 54.4%), only 8.9% reported mental health services use. Overall, mental health service use was not significantly associated with mental illness-related stigma, F(1, 14568) = 1.27, p = .259, η_p_^2^ = .000. However, a significant interaction was seen between mental health service use and sex, F(1, 14569) = 8.69, p = .004, η_p_^2^ = .001. Simple slopes analysis indicated that sex moderated the relationship between mental health services use and stigma, R^2^ = .008, F(10, 14568) = 11.21, p <.001, where mental health service use was associated with greater stigma among female students (p <.001), but not among male students (p = .527).

**Conclusions:**

These findings indicate that a substantial treatment gap for depressive symptoms persists among adolescents and students in Chongqing, China. Notably, among female students, mental health service use in the past 12 months was associated with heightened mental illness-related stigma. This underscores the need for interventions that specifically address stigma in the context of mental health service use.

## 1 Introduction

Concerns regarding adolescent and early youth mental health have continued to rise following the COVID-19 pandemic (1). Recent estimates suggest that the prevalence of depressive symptoms in this population ranges between 26.17% and 31% in China (2, 3). However, despite this increasing prevalence, little is known about the extent to which adolescents and early youth seek or access mental health services.

Previous studies on the mental health treatment gap have primarily focused on depressive disorders among adults. Accordingly, the treatment gap for Chinese adults with depressive disorders was 90.5% (4). A limitation to this study, however, is that it was conducted during the COVID-19 pandemic, when the medical system was under significant strain. Beyond this evidence, no research to date has examined the treatment gap for depressive symptoms among Chinese adolescents and early youth in the post-pandemic context.

Recent studies have sought to address the mental health treatment gap by identifying factors contributing to the decreased use of mental health services. One of the most critical factors is mental illness-related stigma (5, 6). Evidence indicates that higher levels of stigma are associated with reduced treatment-seeking behaviour (7). In response, numerous interventions have been developed to reduce mental illness-related stigma. However, some criticisms argue that the mainstream psychoeducation and the clinical disclosure of mental illness, particularly those emphasising biological explanations, could be iatrogenic, as they risk reinforcing rather than reducing stigma (8, 8). In China, mental health services often rely on relevant biologically based explanatory models.

To date, little evidence has explored the relationship between receiving mental health services and the mental health-related stigma that results from such services, or whether the relationship is moderated by individual differences, such as sex and ethnicity. The current study aims to investigate the treatment gap of depressive symptoms among Chinese adolescents and early youth. Specifically, it examines the relationship between receiving mental health services and personal mental illness-related stigma, as well as the moderating effects of sex and ethnicity.

## 2 Method

### 2.1 Participants

An online cross-sectional survey was conducted among students from 23 secondary schools, 14 high schools and 5 universities/colleges in Chongqing, China (including four minority-autonomous regions). Data was collected between November 2024 to February 2025 using a convenience sampling method.

The inclusion criteria were: (1) students aged between 11 and 24, and (2) students studying and residing in Chongqing. Students who self-reported any clinically diagnosed mental health disorder or psychiatric condition were excluded. Participation was voluntary, and no remuneration was provided.

### 2.2 Procedure

Educational institutions in Chongqing were first contacted, and permission was obtained to distribute the survey. The survey was hosted on the “*Wenjuanxing*” platform and accessed through a QR code. After reading a brief introduction to the study, the students provided informed consent to participate. The survey included demographic questions, measures of depressive and anxiety symptoms, mental health service use within the past 12 months, and personal stigma towards mental illness.

### 2.3 Measurements

#### 2.3.1 Demographic information

Students reported their (1) date of birth, (2) sex, (3) ethnicity, (4) family income level, (5) parental education level, and (6) mental health history. Date of birth was entered in an open field and used to calculate age. Sex was reported on a binary “Male/Female” scale. Ethnicity was selected from a pull-down menu. Family income was reported on a three-point ordinal scale: “≤ ¥4999/month”, “¥5000–¥19999/month,” and “≥ ¥20000/month”. Mental health history was assessed with the question: “Have you been diagnosed with any mental health disorder or psychiatric condition in the past 12 months?”, reported on a binary “Yes/No” scale, with an open-text field for specifying the condition if applicable.

#### 2.3.2 Depressive symptoms

Depressive symptoms were measured using the Patient Health Questionnaire-9 [PHQ-9; (9)], which has been validated among Chinese adolescents and youth (Cronbach’s σ = .84) (10). A score of ≥ 5 was used to indicate the presence of depressive symptoms.

#### 2.3.3 Anxiety symptoms

Anxiety symptoms were assessed using the Generalised Anxiety Disorder Scale-7 [GAD-7; (11)], which has also been validated among Chinese adolescents and youth (Cronbach’s σ = .94) (12). A score of ≥ 5 was used to indicate the presence of anxiety symptoms.

#### 2.3.4 Mental health service use

Service use was assessed with the question: “Have you received any mental health care services in the past 12 months?”, with responses recorded on a binary “Yes/No” scale.

#### 2.3.5 Mental illness-related stigma

Mental illness-related stigma was assessed using the Perceived Devaluation-Discrimination Scale [PDD; (13)], which has been validated in Chinese populations (Cronbach’s σ = .86) (14).

### 2.4 Statistical analysis

Descriptive statistics were calculated to summarise participants’ demographic characteristics and rates of mental health service use in the overall sample and among students with depressive symptoms. ANOVAs were conducted to examine the main effect of mental health service use on stigma scores among students with depressive symptoms. Interaction terms were included to test whether sex or ethnicity moderated this relationship, while controlling for demographic covariates. Where significant interactions emerged, exploratory moderation analyses were conducted, followed by simple slopes analyses to probe moderating effects.

### 2.5 Ethics

The study protocol was approved by the Biomedical Research Ethics Committee of West China Hospital, Sichuan University (No: 2022-1790).

## 3 Results

### 3.1 Participants

A total of 33,409 adolescents and early youth attending schools in Chongqing completed the survey (mean age: 16.27 ± 2.45). Detailed demographic characteristics for the full sample are presented in **Table 1**, and those for students reporting depressive symptoms are presented in **Table 2**. Overall, 43.6% reported depressive symptoms (PHQ-9 ≥ 5, N = 14579), with only 8.9% receiving mental health services within the past 12 months. No statistical difference was observed in mental health service use between female (9.1%) and male (8.6%) students, Chi (χ^2^) = .975, p = .334, V = .008. However, a significant difference emerged between Han Chinese students (9.7%) and students of other minority ethnic groups (8.4%), Chi (χ^2^) = 6.751, p = .010, V = .022, though the effect size was small.

**Table 1:** Overall sample characteristics information.

Items		Mean (SD)	Range
Age		16.27 (2.45)	12–23
		N	%
Sex	Male	16832	50.4
	Female	16577	49.6
Ethnicity	Han Chinese	14749	44.1
	Minority	18660	55.9
Grade	Secondary School	11306	33.8
	High School	10258	30.7
	Higher Education	11845	35.5
Father’s Education	Primary School	8094	24.2
	Secondary School	16659	49.9
	High School	5984	17.9
	Higher Education	2672	8.0
Mother’s Education	Primary School	11581	34.7
	Secondary School	14729	44.1
	High School	5010	15.0
	Higher Education	2089	6.3
Family Income	≤¥4999/m	12814	38.4
	¥5000–¥19999/m	20147	60.3
	≥¥20000/m	448	1.3
Depressive Symptoms	PHQ-9 ≥ 5	14579	43.6

**Table 2:** Characteristics information of students with depressive symptoms.

Items		Mean (SD)	Range
Age		15.95 (2.21)	12–23
		N	%
Sex	Male	6650	45.6
	Female	7929	54.4
Ethnicity	Han Chinese	5165	35.4
	Minority	9414	64.6
Grade	Secondary School	5112	35.1
	High School	5934	40.7
	Higher Education	3533	24.2
Father’s Education	Primary School	3776	25.9
	Secondary School	7306	50.1
	High School	2434	16.7
	Higher Education	1063	7.3
Mother’s Education	Primary School	5309	36.4
	Secondary School	6426	44.1
	High School	2027	13.9
	Higher Education	817	5.6
Family Income	≤¥4999/m	6268	43.0
	¥5000–¥19999/m	8110	55.6
	≥¥20000/m	201	1.4
Depressive Symptoms	Depressive Symptoms Only	3051	20.9
	Depressive Symptoms with Anxiety Symptoms	11528	79.1
Mental Health Service Use	Yes	1291	8.9

### 3.2 Main effect of mental health service use on mental illness-related stigma

An ANOVA was conducted to examine the association between mental health service use in the past 12 months and mental illness-related stigma, controlling for sex, ethnicity, grade level, family income, parental education, and anxiety symptoms. No significant main effect of mental health service use was observed, F(1, 14568) = 1.27, p = .259, η_p_^2^ = .000. Similarly, no significant effects were found for sex, F(1, 14568) = .29, p = .590, η_p_^2^ = .000, ethinicity, F(1, 14568) = .33, p = .563, η_p_^2^ = .000, grade level, F(1, 14568) = 1.07, p = .302, η_p_^2^ = .000, father’s education, F(1, 14568) = 2.57, p = .133, η_p_^2^ = .000, or mother’s education, F(1, 14568) = .00, p = .993, η_p_^2^ = .000. Small but significant effects were observed for family income, F(1, 14568) = 27.22, p <.001, η_p_^2^ = 002, and anxiety symptoms, F(1, 14568) = 40.88, p <.001, η_p_^2^ = .003.

No significant interaction between mental health service use and ethnicity was observed, F(1, 14569) = .01, p = .932, η_p_^2^ = .000. However, a significant interaction by sex was found, F(1, 14569) = 8.69, p = .004, η_p_^2^ = .001. Follow-up analyses stratified by sex suggest a significant main effect of mental health service use on mental illness-related stigma among female students, F(1, 7927) = 25.59, p <.001, η_p_^2^ = .003, but not among male students, F(1, 6648) = .83, p = .363, η_p_^2^ = .000. These results suggest that sex may moderate the relationship between receiving mental health services and mental illness-related stigma, whereas ethnicity does not appear to have a moderating effect.

### 3.3 Exploratory moderation analysis

**Figure 1:**
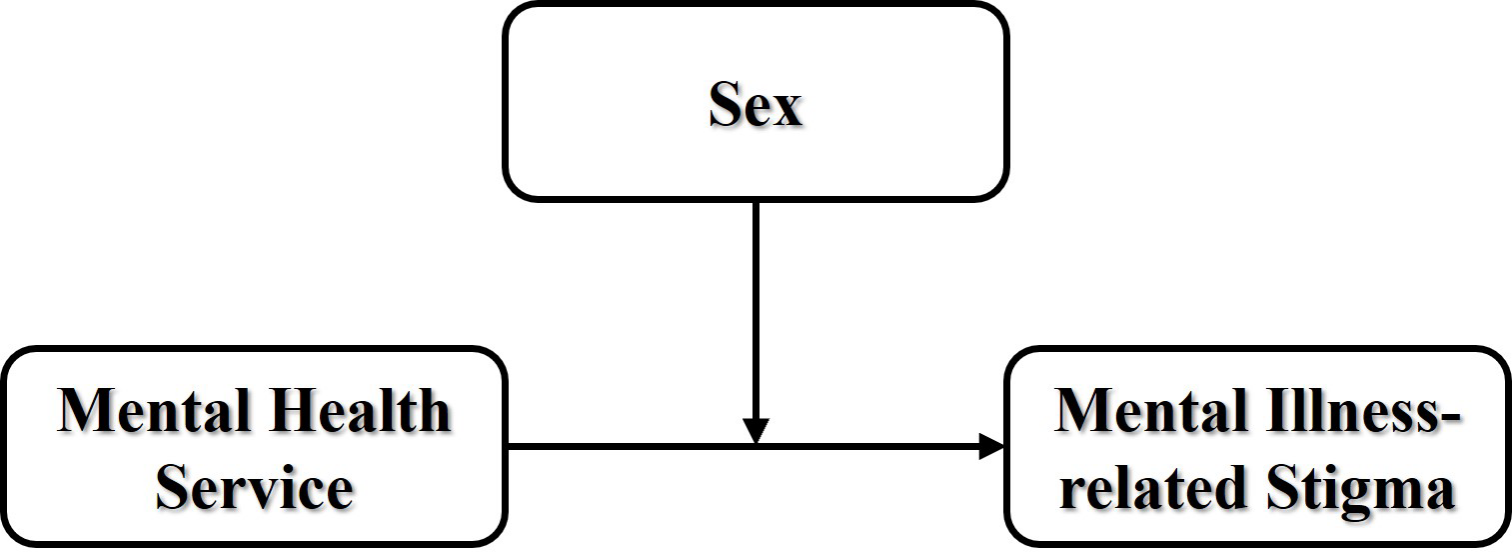
Moderation model of sex moderating the association between receiving mental health services and mental illness-related stigma.

Mental health service use in the past 12 months, mental illness-related stigma, and student sex were entered into a moderation model for simple slopes analysis, controlling for age, ethnicity, grade, parental education, family income and anxiety symptoms ratings. As shown in **Figure 1**, the overall moderation model was significant, R^2^ = .008, F(10, 14568) = 11.21, p <.001, with a significant interaction between sex and stigma, F(1, 14568) = 8.71, p = .003. Among the covariates, only family income (p <.001) and anxiety symptoms (p <.001) were significant.

**Figure 2:**
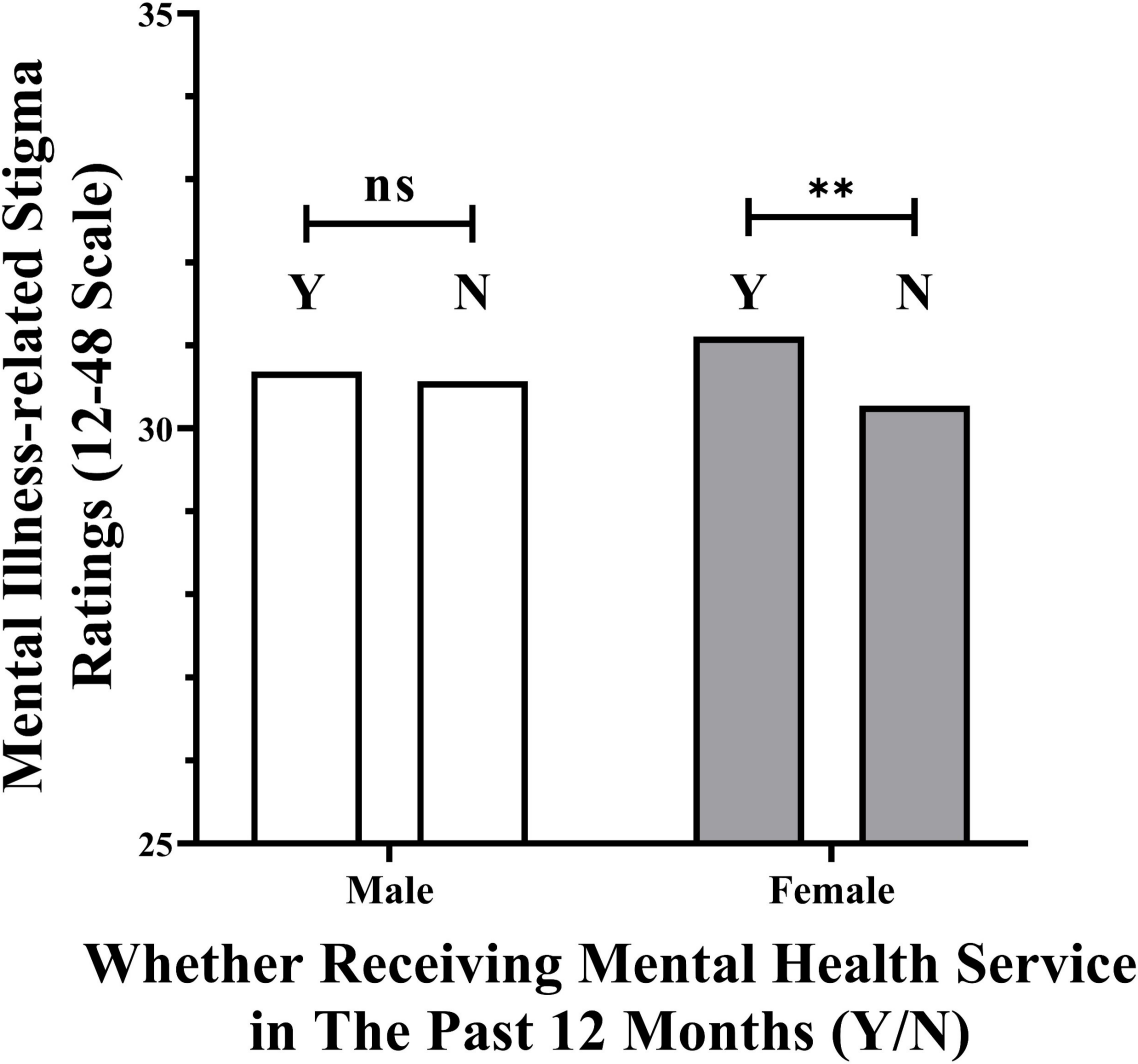
Associations between mental health service use and mental illness-related stigma.

As shown in **Figure 2**, the association between mental health service use and mental illness-related stigma was significant among female students, β = −.83, SE = .16, t = −5.13, p <.001, but not among male students, β = −.12, SE = .18, t = −.63, p = .527. These results suggest that female students may be more affected by mental illness-related stigma following mental health use in the past 12 months compared to male students.

## 4 Discussion

The current study conducted a cross-sectional survey among adolescents and early youth students in Chongqing, China, to investigate their use of mental health services in 2024 and mental illness-related stigma. Results showed that 43.6% students reported depressive symptoms, among which 79.1% also reported co-occurring anxiety symptoms. This could be caused by the increased academic pressure among Chinese students (15). However, only 8.9% of students with depressive symptoms reported using mental health services in the past 12 months, leaving a treatment gap of 91.1%. No significant sex differences were observed in treatment gap rates. However, ethnic minority students reported a larger gap (100% − 8.4% = 91.6%) compared to Han Chinese students (100% − 9.7% = 90.3%). In addition, a significant association between mental health service use and mental illness-related stigma was found among female students but not among male students.

The first major finding was the substantial treatment gap in mental health services (91.1%) among adolescents and early youth students in Chongqing who reported depressive symptoms. To the best of the authors’ knowledge, this is among one of the first studies to report on the mental health treatment gap in Chinese adolescent and early youth students, as most prior research has focused on Chinese adults or population samples from other regions (4, 16). Previous studies have estimated that only 9.5% of Chinese adults with depressive disorders received treatment, corresponding to a treatment gap of 90.5%. The comparable findings across adolescents, early youth, and adults underscore an urgent need to expand access to mental health services in China. On the one hand, this could imply some lack of medical resources in mental health and psychiatry in the healthcare system. On the other hand, this could also be caused by mental health-related stigma of the students and their family members (7). Although treatment gap rates did not differ significantly between male and female students, the small but significant difference between Han Chinese and ethnic minority students highlights the importance of outreach within minority communities.

The second key finding revealed a sex-based difference, where a stronger association between mental service use and mental illness-related stigma emerged among female students but not among male students. Although the current study used a cross-sectional survey, this association is not expected to be dual-directional. Because stigma was assessed at the time of the survey and service use referred to the preceding 12 months, this finding is best interpreted as stigma that follows after service use rather than stigma that predicts service use. A potential explanation for the observed effect relates to the nature of psychoeducation and clinical disclosure within current interventions. Many mainstream mental health programs emphasise biological explanations of mental illness, framing it as a brain disease. Although intended to reduce stigma, this framing can have unintended negative consequences, such as fostering pessimism about recovery or reinforcing self-stigma (8, 17). For instance, in a recent randomised controlled trial, hazardous and dependent alcohol drinkers from the UK who received psychoeducation material framing substance abuse as a compulsive brain disorder showed a reduction in confidence in quitting drinking compared with those who received psychosocial or neutral materials (18). Such iatrogenic effects after a certain type of mental health intervention seemed to be universal across culture and nationality. These findings highlight that interventions aiming to reduce stigma must be carefully designed, and that re-evaluating communication strategies within mental health services may be critical.

The observed sex difference aligns with prior research showing that female adolescents are more responsive to psychoeducation. For example, in a randomised controlled trial of a depression education program among adolescents, it was found that female participants demonstrated greater rates of depression literacy than male participants after receiving the psychoeducation program (19). Taken together, prior evidence and the current results suggest that female students may be more influenced by psychoeducation and clinical disclosure during the mental health service they received. Accordingly, tailoring psychoeducation content and clinical disclosure should aim to minimise these unintended stigma effects, particularly for female students. Ongoing research could further clarify how specific psychoeducational strategies shape adolescents’ perceptions of mental illness, thereby informing improvements in mental health services.

Several limitations should be noted. First, the cross-sectional design does not imply causal effects. Although the study design assessed mental health service use in the past year and stigma at the time of the survey, baseline stigma levels and other potential confounding factors were not controlled. Longitudinal designs will be needed to establish a causal relationship. Second, the current study included only adolescents and early youth who were currently enrolled in school, not including peers who had dropped out or were not pursuing education. This limitation is noteworthy, as these populations may be at even greater risk. Therefore, the generalisability of the findings should be interpreted with caution.

Despite these limitations, the current study has important implications. It is among the first to document the mental health treatment gap among Chinese adolescents and early youths with depressive symptoms, providing a valuable reference for public health policy. Moreover, the findings highlight a concerning link between receiving mental health services and mental illness-related stigma, specifically among female students, underscoring the need to evaluate psychoeducational approaches and clinical disclosure practices for the healthcare system. Future work should prioritise stigma-reducing interventions and improved communication within mental health services to ensure that treatment supports recovery while actively working to dismantle stigma.

In conclusion, the current study conducted a cross-sectional survey to investigate the treatment gap of depressive symptoms and the relationship between mental health-related stigma among adolescents and early youth students in Chongqing, China. There was a substantial treatment gap rate among students, and mental health-related stigma increased with receiving mental health services among female students. These results provided insights for the healthcare system to evaluate the existing mental health service.

## Data Availability

The raw data supporting the conclusions of this article will be made available by the authors, without undue reservation.
